# Research trends on alphavirus receptors: a bibliometric analysis

**DOI:** 10.3389/fcimb.2024.1388360

**Published:** 2024-05-22

**Authors:** Runqi Chen, Zirui Wang, Leiliang Zhang

**Affiliations:** ^1^ Department of Clinical Laboratory Medicine, The First Affiliated Hospital of Shandong First Medical University & Shandong Provincial Qianfoshan Hospital, Jinan, Shandong, China; ^2^ Department of Pathogen Biology, School of Clinical and Basic Medical Sciences, Shandong First Medical University & Shandong Academy of Medical Sciences, Jinan, Shandong, China; ^3^ Medical Science and Technology Innovation Center, Shandong First Medical University & Shandong Academy of Medical Sciences, Jinan, Shandong, China

**Keywords:** bibliometrics, alphavirus receptor, viral entry, CiteSpace, research trend

## Abstract

**Background:**

Alphaviruses are a diverse group of pathogens that have garnered considerable attention due to their impact on human health. By investigating alphavirus receptors, researchers can elucidate viral entry mechanisms and gain important clues for the prevention and treatment of viral diseases. This study presents an in-depth analysis of the research progress made in the field of alphavirus receptors through bibliometric analysis.

**Methods:**

This study encompasses various aspects, including historical development, annual publication trends, author and cited-author analysis, institutional affiliations, global distribution of research contributions, reference analysis with strongest citation bursts, keyword analysis, and a detailed exploration of recent discoveries in alphavirus receptor research.

**Results:**

The results of this bibliometric analysis highlight key milestones in alphavirus receptor research, demonstrating the progression of knowledge in this field over time. Additionally, the analysis reveals current research hotspots and identifies emerging frontiers, which can guide future investigations and inspire novel therapeutic strategies.

**Conclusion:**

This study provides an overview of the state of the art in alphavirus receptor research, consolidating the existing knowledge and paving the way for further advancements. By shedding light on the significant developments and emerging areas of interest, this study serves as a valuable resource for researchers, clinicians, and policymakers engaged in combating alphavirus infections and improving public health.

## 1 Introduction

Alphaviruses are a group of globally insect-transmitted RNA viruses that belong to the *Togaviridae* family (Weaver et al., 2012). The alphaviruses consist of 29 different species of positive-strand RNA viruses that cause a wide variety of diseases in humans, domesticated and wild terrestrial vertebrates, as well as fish (Weaver et al., 2012). Two clinical syndromes occur in humans with alphavirus infections: acute encephalitis and neurological diseases caused by Venezuelan equine encephalitis virus (VEEV), Eastern equine encephalitis virus (EEEV), and Western equine encephalitis virus (WEEV); and acute and chronic musculoskeletal disease and arthritis caused by *Chikungunya virus* (CHIKV), *Ross River virus* (RRV), *Barmah Forest virus* (BFV), *O’nyong’nyong virus* (ONNV), *Mayaro virus* (MAYV), and *Sindbis virus* (SINV) (Zimmerman et al., 2023a). The New World viruses, including VEEV and EEEV, evolved separately from those of the Old World, including SINV and *Semliki Forest virus* (SFV) (Garmashova et al., 2007). The WEEV is a group of arthropod-borne viruses in the alphavirus family. All New World viruses in the WEE complex virus, except for the *Aura virus*, are recombinants derived from EEEV and *Sindbis*-like viruses. The Old World members of the WEE complex virus did not appear to have recombinant genomes (Weaver et al., 1997). Chikungunya fever (CHIKF) was first described in 1952 following an outbreak on the Makonde Plateau, along the border between Tanganyika and Mozambique. From 2006 onward, CHIKF has emerged, even in nonendemic areas, as an important disease among returning travelers (Silva and Dermody, 2017). CHIKV returned to the American tropics in 2013 (Halstead, 2015).

The alphavirus RNA genome is an 11.5-kb, single-stranded RNA of positive polarity that encodes only a few proteins and contains a 5′-methylguanylate cap and a 3′-polyadenylate tail (Strauss et al., 1984). The genome is directly translated into the viral nonstructural proteins nsP1, nsP2, nsP3, and nsP4, and six structural proteins: capsid, E1, E2, E3, 6K, and TF (Snyder et al., 2013; Kim et al., 2021). The nonstructural proteins are required for virus replication, protein modification, and immune evasion. NsP1 of the alphavirus CHIKV is responsible for RNA capping, inducing migrasome formation, and membrane binding of the replication machinery (Zhang et al., 2021, 2022). Nsp2 acts as a helicase and a RNA protease (Law et al., 2019). Nsp3 functions to form replication complexes for negative-strand RNA (Wang et al., 1994). Nsp4 strongly influences efficient subgenomic RNA synthesis and determines the template RNA preference and efficiency of RNA synthesis (Lello et al., 2021). The structural proteins are synthesized from a subgenomic promoter and cleaved co- and posttranslationally (Basore et al., 2019). E2 and E1 are transmembrane proteins that interact to form a heterodimer, and recent analysis suggests that both E2 and E1 proteins contribute to receptor engagement (Zimmerman et al., 2023a). E2 consists of three domains, labeled A, B, and C. On the other hand, E1 is a class II fusion protein with three domains: DI, DII, and DIII. Upon trimerization, the E1 DI and DII domains fold into a hairpin-like structure (Holmes et al., 2020). The E3 protein is essential for the proper folding of p62 and the formation of the p62-E1 heterodimer. E1 monomers lie at the base of the surface spikes and form trimers surrounding the icosahedral axes, while E2 localizes to a long, thin, leaf-like structure on the top of the spike (Zimmerman et al., 2023a). There are nine receptors known for alphaviruses: laminin receptor, natural resistance-associated macrophage protein (NRAMP)/NRAMP2, prohibition 1 (PHB1), CD147 protein complex, matrix remodeling associated 8 (MRXA8), low-density lipoprotein receptor class A domain containing 3 (LDLRAD3), low-density lipoprotein receptor (LDLR), very low-density lipoprotein receptor (VLDLR), and apolipoprotein E receptor 2 (ApoER2) (Zimmerman et al., 2023a). EEEV interacts with the ligand-binding domains of VLDLR and ApoER2. (Clark et al., 2022) The E2 and E1 glycoproteins of alphaviruses interact with their receptors. Entry into evolutionarily divergent host cells can be accomplished by the recognition of different cellular receptors in different species.

Bibliometric analysis is a statistical method based on public literature databases that provides a quantitative and qualitative evaluation of publications to aid in the analysis of search hotspots and trends in a specific field. We used literature analysis software to search for potent hotspots in the field of alphavirus receptors. Bibliometrics is a study method that quantifies the impact of independent research findings on the development of a specific virus field. To our knowledge, no publications related to alphaviruses that apply bibliometrics have been found before. Hence, this study explored the major events in this field over the past decade. Bibliometric analysis uses statistics to describe the tendency of scientific viruses. This type of research summarizes the trends in specific disciplines and provides new insight into the future development of alphavirus receptors. In this study, we highlight advances in the field of alphavirus receptors using the bibliometric study method.

## 2 Methods

**Figure 1:**
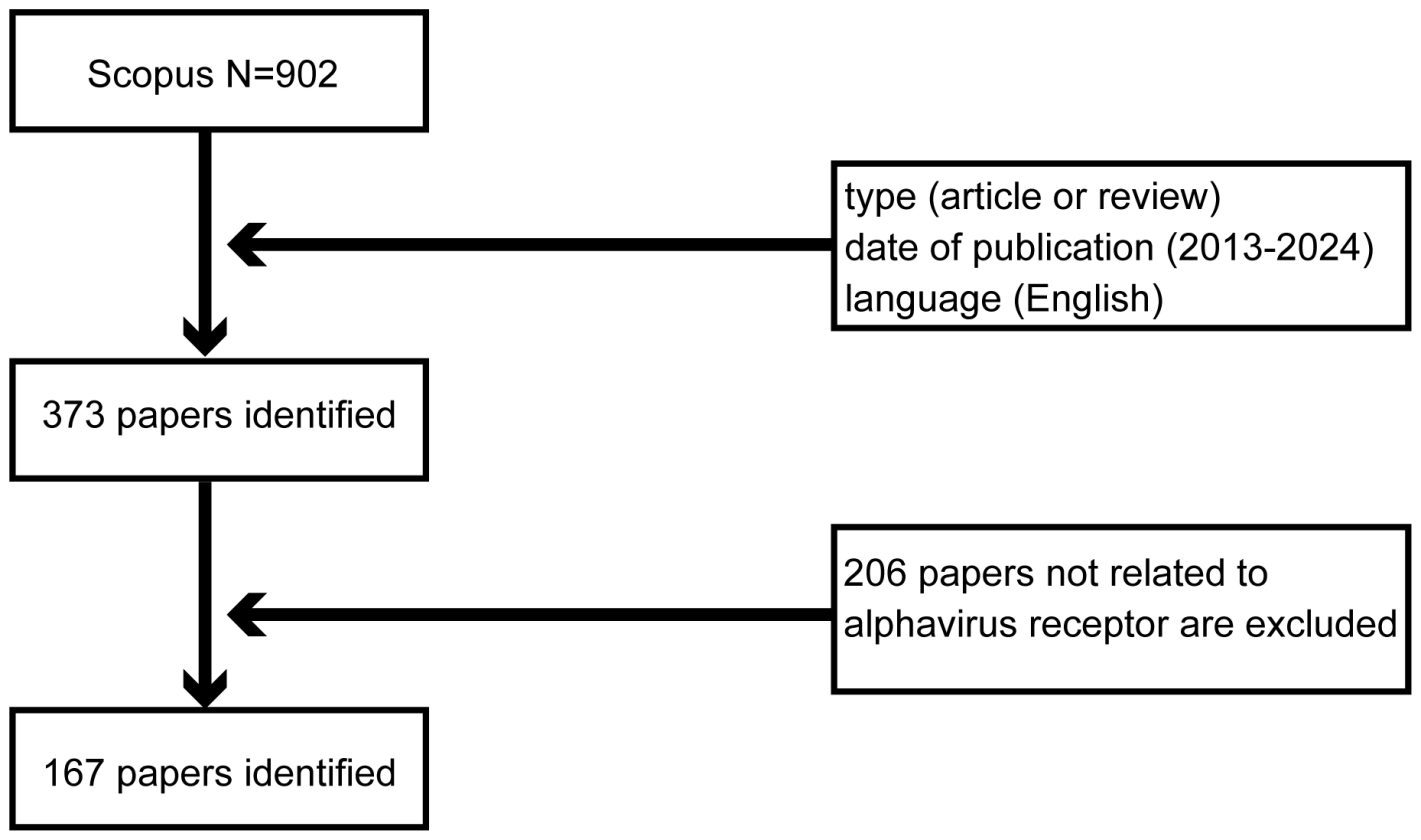
Flowchart of the literature-searching strategy used in this study.

Scopus is a globally renowned and high-impact academic repository that covers articles across a wide range of fields. On 12 January 2024, a comprehensive search was conducted on Scopus (http://www.scopus.com/) to find articles related to the alphavirus receptor. The initial search for “alphavirus receptor” yielded 902 records. To refine the selection, we established criteria based on research objectives, topic relevance, publication period, and other characteristics. Only articles and reviews written in English and published between 2013 and 2024 were included, resulting in a reduced number of 373 records. Special types of publications, such as letters, conference papers, and notes that did not match the study theme, were excluded. Finally, 167 studies were exported in CSV format after excluding studies unrelated to the alphavirus receptor. A flow-process diagram provides an illustration of the selection process (**Figure 1**).

To better analyze the characteristics of these articles and reviews, a series of methods were employed. CiteSpace (version 6.2. R7) was used to construct a hotspot burst map based on its special functions. CiteSpace is a Java-based application that analyzes and visualizes hotspots and research frontiers in scientific literature within a specific discipline or knowledge domain over a certain period of time. It employs metrology, co-occurrence analysis, and cluster analysis (Chen, 2004, 2006). The map helps us better understand this research. Each node on the graph represents an author, a country, or a keyword and can be divided into several clusters according to their attributes. The size of the node represents the number of publications, and the thickness of the connection between the nodes reflects the level of cooperation (Pei et al., 2022).

Additionally, CiteSpace was used to measure centrality, a metric that defines the importance of network nodes. More prominent nodes represent higher centrality, meaning they have more connections within the network passing through them (Han et al., 2023). Another bibliometric tool called “Bibliometrix” was applied, following the logical workflow of classical bibliometrics and combining statistical calculations and visualizations. The Bibliometrix software package utilizes the statistical and tracing capabilities of the R language (Cui et al., 2022). This online analysis platform helps to display country cooperation on the map and generate keyword cloud maps. These tools were employed to visualize and analyze the characteristics of articles, identify the current research focus, and identify potential trends in the alphavirus receptor field (Li et al., 2023).

The VOS viewer, a literature analysis and knowledge visualization software developed by Leiden University in the Netherlands, was used in conjunction with Scimago Graphica to create a chord diagram. This diagram helps to clearly demonstrate the relationship between publication volume and cooperation among different countries and regions (van Eck and Waltman, 2010).

## 3 Results

### 3.1 Annual publications

**Figure 2:**
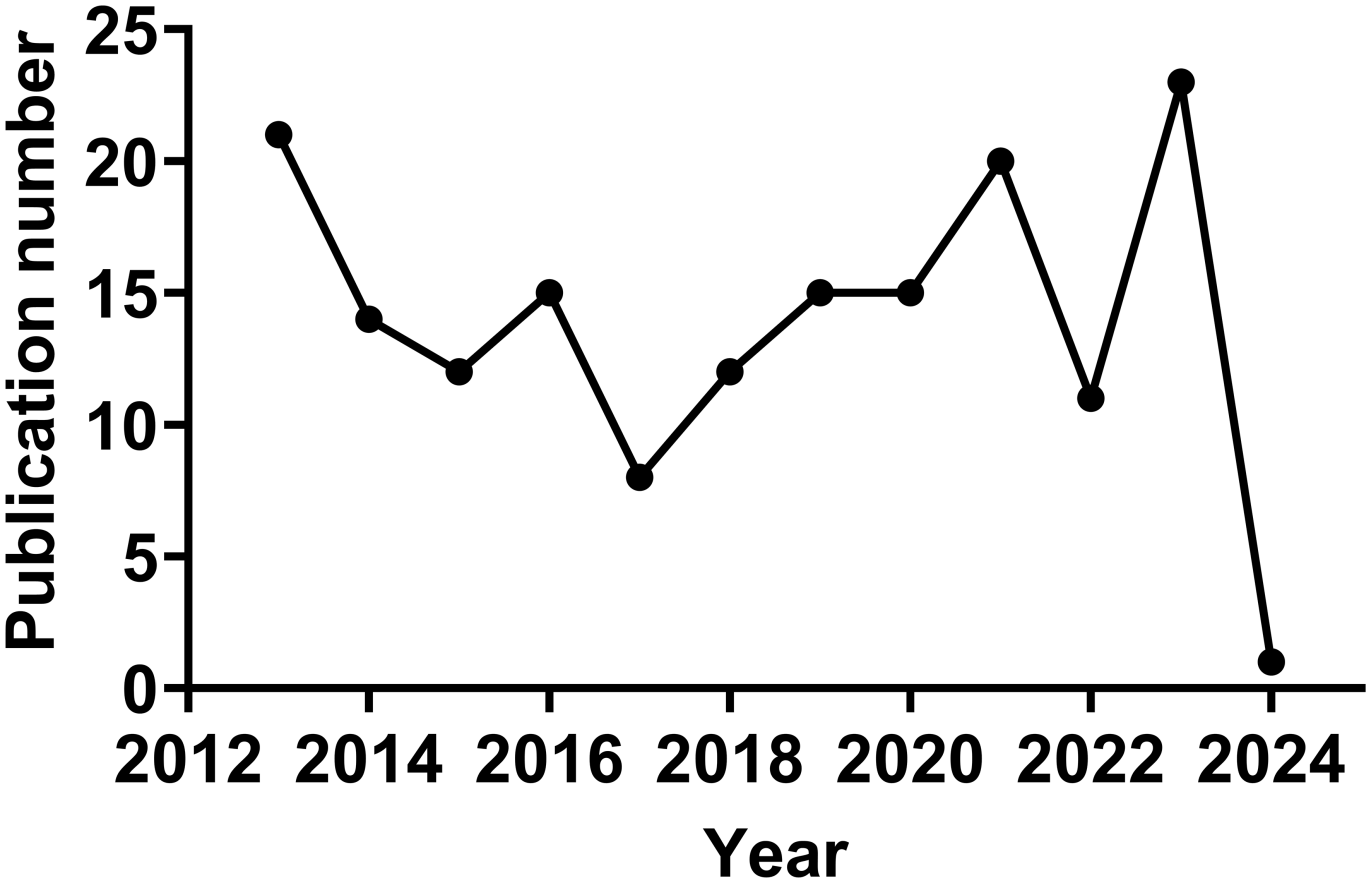
Trends in the literature on alphavirus receptors over the past 12 years.

In the Citespace analysis of alphavirus receptors from 2013 to 2024, a total of 167 publications were retrieved. The annual number of articles is shown in **Figure 2**. The number of publications can be divided into three phases. The first phase is from 2013 to 2017 and presents a general downward trend, except for a slight increase in 2016. Notably, the number in 2013 reached a high point (*n* = 21), partly due to the 2012 outbreak of the East/Central/South African strain of CHIKV in Papua New Guinea and the spread of CHIKV to the Western Hemisphere (Zimmerman et al., 2023a). The number of studies reached its lowest point in 2017 (*N* = 8). The second phase is from 2017 to 2021 and shows a trend of continuous growth. The number in 2021 also reaches a high point (*n* = 20). The third phase is from 2022 to 2024, with a high level maintained in 2021 and 2023 and an additional article in the year 2024. Each trend may represent an outbreak of a specific alphavirus or new research on the receptor structure of the viruses and the host range of alphaviruses.

### 3.2 Author and cited-author analysis

**Figure 3:**
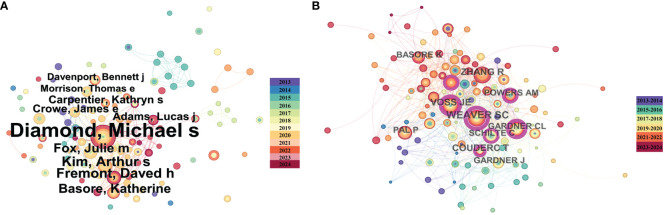
Co-author and high-cited author analysis of the literature on alphavirus receptor research. **(A)** Co-author network map in the field of alphavirus receptor, with the size of the circle representing the number of publications and the link color showing the degree of cooperation among them. **(B)** High-cited author network map in the same field, with the size of the circle representing the number of cited publications and the link color showing the degree of cooperation among them.

The publication of authors and their co-authorship networks can help identify outstanding scholars leading research in the field and provide information on collaboration among authors, which is crucial for future research development. We analyzed publications using a time-slicing of 1 year, resulting in a total of 270 authors involved in alphavirus receptor research. Each node in **Figure 3A** generated using Citespace (6.2. R7) represents an author, with the circle’s size indicating the number of articles published by the author. Diamond published the most papers (*n* = 26), followed by Kim (*n* = 8), Fremont (*n* = 8), Basore (*n* = 7), and Fox (*n* = 7). **Figure 3A** also illustrates the authors’ collaboration and contribution through the node size and link color depth. Diamond had the highest link strength, and their research collaborations appear to be steady and close. We then analyzed cited authors using a time-slicing of 2 years, selecting the top 50 levels per slice. Co-citation networks often have a vast number of links, and displaying links indiscriminately can cause clustering. According to citation analysis in **Figure 3B** generated using Citespace (6.2. R7), the most cited author was Weaver (*n* = 55), followed by Zhang (*n* = 45), Voss (*n* = 42), and Coudert (*n* = 37). It is apparent that many authors tend to collaborate with a relatively stable group of collaborators, resulting in several major author clusters typically consisting of two or more authors.

### 3.3 Institutional analysis

**Figure 4:**
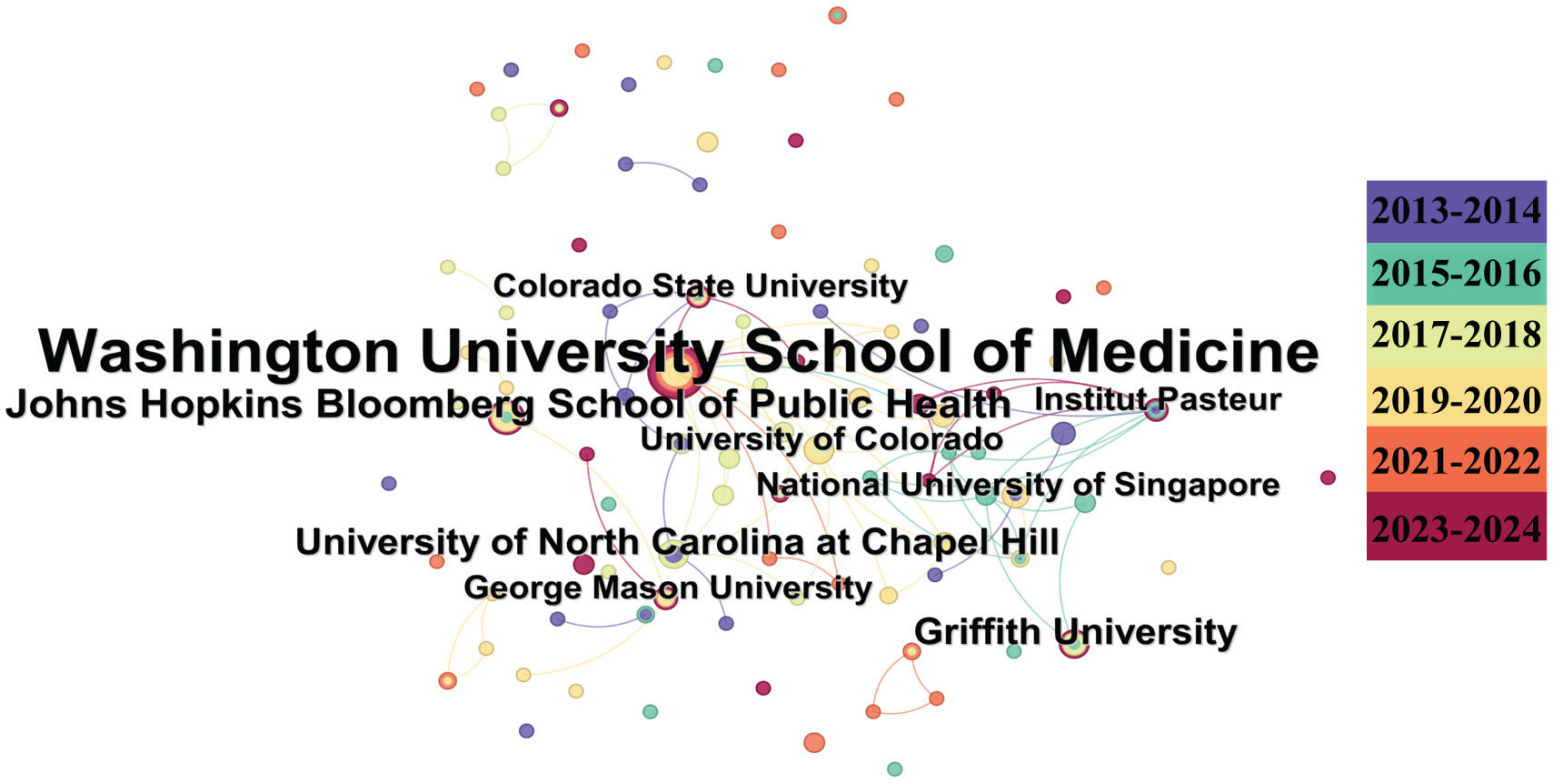
The institutional analysis of the literature on alphavirus receptor research. Institutions involved in the field of alphavirus receptors, with the size of the circle representing the number of publications and the link color showing the degree of cooperation among them.

The institutional analysis runs over a time-slicing of 2 years. The collaboration map in **Figure 4** illustrates the level of collaboration among these institutions. Washington University School of Medicine (*n* = 19), Johns Hopkins Bloomberg School of Public Health (*n* = 6), Griffith University (*n* = 5), and the University of North Carolina at Chapel Hill (*n* = 5) are the most prominent institutions in the collaboration map, indicating their significant role in this field.

### 3.4 Distribution of countries

**Figure 5:**
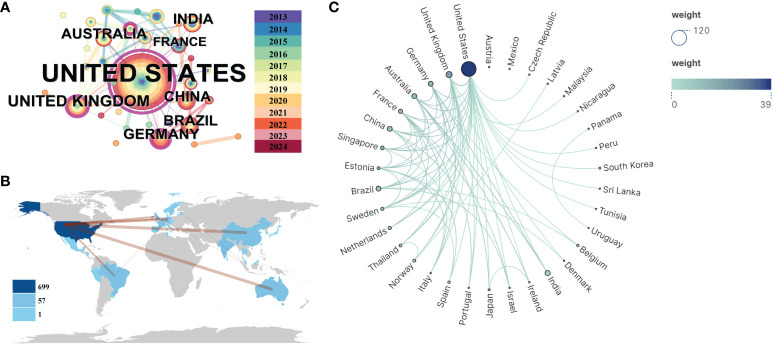
Visualization and analysis of international collaboration networks in alphavirus receptor research. **(A)** Co-network map of countries, with the size of the circle representing the number of publications and the link color showing the degree of cooperation among them. **(B)** Cooperation network of countries in a circular view, with the size and shade of the circle representing the number of publications and the number of lines representing the amount of cooperation. **(C)** Country collaboration map related to alphavirus receptors during the considered period. The color depth in the map represents the number of publications produced by each country, while the thickness of the connecting lines indicates the level of cooperation between countries. A thicker connecting line indicates a higher degree of collaboration between two countries in the field of alphavirus receptors. This map provides insights into the collaborative relationships and networks established among different countries in advancing research on alphavirus receptors.

An analysis of national publication counts reveals the countries/regions that have made the most contributions in this field. **Figure 5A** generated using Citespace (6.2. R7), **Figure 5B** analyzed by VOS viewer, and **Figure 5C** conducted by bibliometrix display the distribution of publications across 33 countries, primarily located in America, South and East Asia, Europe, and Australia. The USA leads with the highest number of publications (*n* = 100), followed by the UK (*n* = 15) and India (*n* = 12). Further analysis of publication numbers was used to construct a collaborative network based on the relationships between countries. Notably, there is extensive cooperation between different countries. The USA, China, Germany, and Australia demonstrate strong collaboration, as indicated by the thickness of the links. The USA, being the largest country, holds a central position in the alphavirus receptor field with a centrality value of 0.84. The UK ranks second with a centrality value of 0.32.

### 3.5 Reference analysis with strongest citation bursts

**Figure 6:**
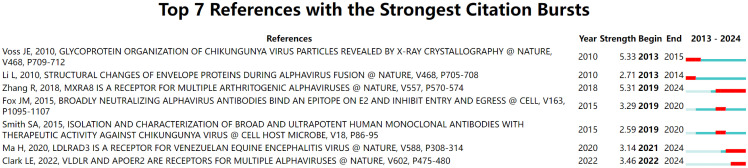
Reference analysis of the top seven references with the strongest citation burst, with the line representing the period of time and the red bar representing strong citation burstiness.

Each line segment in **Figure 6** represents a period of time, and the red bar represents strong citation burstiness. The reference with the first citation burst is “Glycoprotein organization of CHIKV particles revealed by X-ray crystallography” (Voss et al., 2010), which gained prominence from 2013 to 2015. This study unveiled the molecular mechanism underlying the CHIKV invasion of susceptible cells, which is facilitated by two viral glycoproteins, E1 and E2 (Voss et al., 2010). The reference with the second citation burst is “Structural changes of envelope proteins during alphavirus fusion” (Li et al., 2010), cited from 2010 to 2014. In this article, the author reported the structure of SINV and the trimeric spike structure at low pH, representing an intermediate stage in the fusion process and elucidating the maturation process (Li et al., 2010). The reference with the third citation burst is “Mxra8 is a receptor for multiple arthritogenic alphaviruses” (Zhang et al., 2018), which has been widely referenced since 2019 and continues to be a topic of interest. With the application of a genome-wide clustered regularly interspaced short palindromic repeats (CRISPR)-Cas9-based screen, they identified the cell adhesion molecule Mxra8 as an entry mediator for multiple arthritogenic alphaviruses, including CHIKV, RRV, and ONNV (Zhang et al., 2018).

### 3.6 Keyword analysis

**Figure 7:**
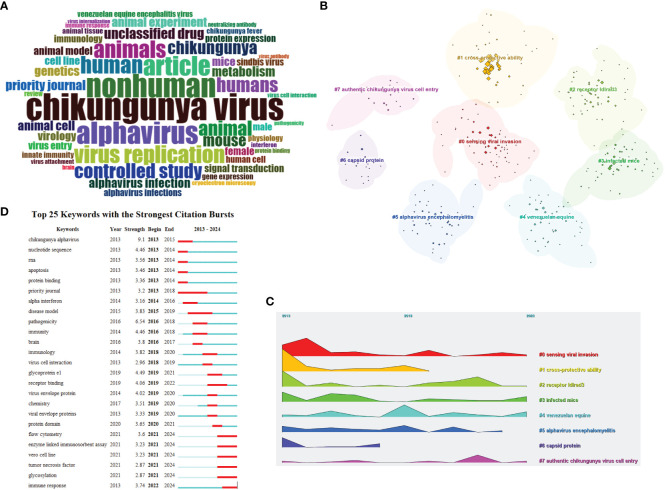
Visualization and analysis of the alphavirus receptor keywords in the literature. **(A)** Word cloud of alphavirus receptors, with different sizes representing the frequency of keywords. **(B)** Cluster view of the keywords, with different colors representing clusters composed of different keywords. **(C)** Landscape view of the keywords, with different clusters, manifested over time. **(D)** Keyword burst detection of the top 25 keywords with the strongest citation burst, with the line representing the period of time and the red bar representing strong citation burstiness.

A word cloud was created by Bibliometrix to visualize the count of author keywords and present a visualization of the words that appear most frequently in research papers (**Figure 7A**). The most common word was “CHIKV”, the second most common word was “nonhuman”, and the third most common word was “alphavirus”. It is evident that receptors in different animal hosts are a key research topic in the field, with a significant portion of research focusing on humans as hosts. The word cloud provides more importance to author keywords that appear more frequently and serves as a visualization method to reveal the known research focus and trends in alphavirus receptor research.

To further investigate the hotspots in this field, keyword analysis was performed using Citespace (6.2.R7). Through cluster calculation, a total of eight clusters were obtained based on the co-occurrence of the keywords (**Figure 7B**). The keyword analysis showed the most occurring keywords related to alphavirus receptor were divided into the following eight clusters: sensing viral invasion, cross-protective ability, receptor ldlrad3, infected mice, Venezuelan equine, alphavirus encephalomyelitis, capsid protein, and authentic CHIKV cell entry. These eight clusters demonstrate the main themes of exploration in this field. The keywords of eight clusters were described along the landscape view (**Figure 7C**) according to the time order, which shows the research progress in the alphavirus receptor field from 2013 to 2024.

The infected mice were considered an important mammal model in the research process on virus receptors. The research process of NRAMP/NRAMP2 involved the mouse model for SINV. As for LDLRAD3, gene editing of mouse ldlrad3 can result in a low viral load in mouse neuronal cells. Next, it was discovered that another receptor, LDLR, was found in mouse neuronal cells. The multifunctional arthritogenic alphavirus capsid protein is crucial for viral infection and has roles in genome encapsulation, budding, and virion assembly. The arthritogenic alphaviral capsid protein can play an important role in vaccine design, can be regarded as a therapeutic target, and can play an important role in the development of diagnosis (Rao and Taylor, 2021). CHIKV can be genetically divided into three lineages: West African; East, Central, and South African; Indian Ocean; and Asian. Data suggested that IOL-based vaccine strains can offer robust cross-protection against strains from other lineages (Langsjoen et al., 2018). Other experiments suggest that antibodies against one of the arthritogenic alphaviruses can cross-react with other group members. However, effective cross-protection would require a higher dose and more vaccinations (Nguyen et al., 2020). The New World alphaviruses, which consist of VEEV, EEEV, and WEEV, induce encephalomyelitis. Cellular infection with alphaviruses can ultimately result in several outcomes. During infection with VEEV, the severity of the disease ranges from asymptomatic to rapid death, with clinically affected equids demonstrating fever and lethargy (Baxter and Heise, 2020).

**Figure 7D** displays the top 25 keywords with the strongest citation bursts. Keywords with citation bursts refer to keywords that are frequently cited by scholars in a specific field over time. **Figure 7D** illustrates each line segment representing a period of time, and the red bar represents strong citation burstiness. Citation bursts for keywords appeared as early as 2013 and as late as 2024. The keyword with the first citation burst is titled “*Chikungunya* alphavirus”. CHIKV is transmitted by arboreal *Aedes* mosquitoes in an enzootic cycle with nonhuman primates as the main reservoir (Vu et al., 2017). Research on the receptors of CHIKV includes PHB1 and MXRA8 (with bovine receptors not belonging to the host range). The keyword with the second strongest citation burst is titled “Nucleotide sequence”, which ended in 2014. The newly strongest citation bursts include virus flow cytometry, enzyme-linked immunosorbent assay, Vero cell line, tumor necrosis factor, glycosylation, and immune response.

### 3.7 An overview of the research progress of the alphavirus receptor

**Figure 8:**
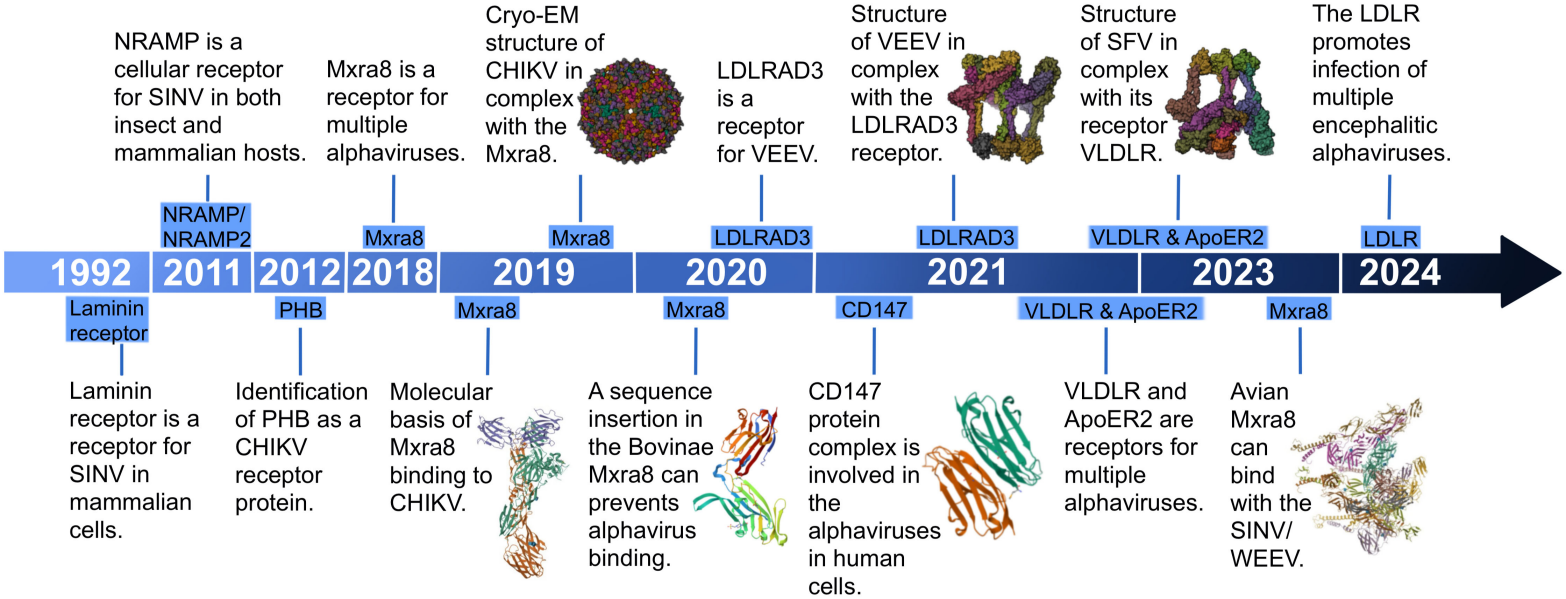
The milestone timeline of alphavirus receptor research. Some relevant research and structure summaries are shown in the timeline. In 2019, the molecular basis of MXRA8 (PDB:6QO8) binding to CHIKV and the cryo-EM structure of CHIKV (PDB: 6NK6) in complex with MXRA8 were found. In 2020, *Bovinae* MXRA8 (PDB:6ORT) was found to be unable to bind with CHIKV. In 2021, CD147 (PDB:3QQN) was found to be involved in the alphaviruses in human cells. The structure of VEEV in complex with LDLRAD3 (PDB:7N1H) was discovered. In 2023, the structure of SFV in complex with its receptor VLDLR (PDB:81HP) was found, and avian MXRA8 (PDB:8EWF) was found to be able to bind with SINV/WEEV.

**Figure 9:**
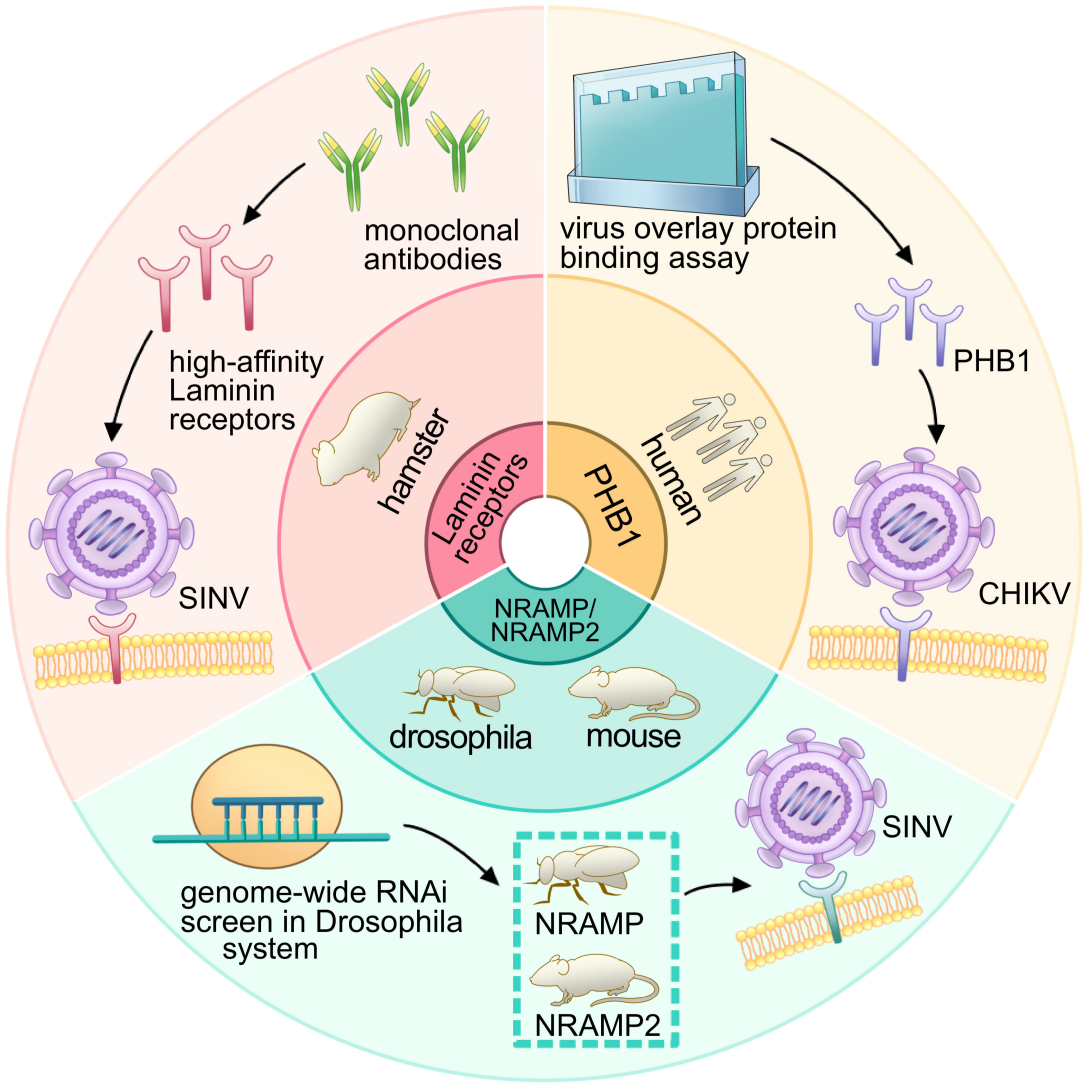
Research process and receptor identification for PHB1, laminin receptor, NRAMP/NRAMP2: insights from experimental approaches. The diagram illustrates the research process involving PHB1, the laminin receptor, and NRAMP/NRAMP2. The inner ring depicts the species of the receptors, while the outer ring represents the corresponding experimental processes. The red panel shows that, under the use of a monoclonal antibody, the high-affinity laminin receptor was regarded as a receptor for SINV in hamsters. The green panel depicts that the genome-wide RNAi screen was used in the *Drosophila* system and identified the NRAMP (*Drosophila*) and NRAMP2 (mouse) as receptors for SINV. The orange panel shows that the virus overlay protein-binding assay was used to identify PHB1 as a receptor for CHIKV in humans.

Experimentally establishing the role of a surface protein as a virus receptor should meet four criteria: direct interaction between the virus and receptors; mediation of virus internalization by the receptor; inhibition of virus infection by neutralizing antibodies against the receptor, soluble receptor decoy molecules, or mutagenesis of the virus receptor binding domain; and correlation between susceptibility of a permissive cell type and receptor expression level (Holmes et al., 2020). Research on alphavirus receptors can be traced back to 1992, when the high-affinity laminin receptor was identified as the first receptor for SINV in mammalian cells (**Figure 8**) (Wang et al., 1992). This receptor, a 67-kDa protein present on the cell surface, binds to basement membrane laminin and plays a role in tumor invasion. Blocking SINV binding to mammalian and mosquito cells was achieved using a monoclonal antibody against the C-terminal domain of the high-affinity laminin receptor (**Figure 9**) (Wang et al., 1992).

In 2011, the second alphavirus receptor, NRAMP/NRAMP2, was discovered as a cellular receptor for SFV in both insect and mammalian hosts (**Figure 8**) (Rose et al., 2011). The NRAMP is crucial for the binding and entry of SINV into *Drosophila* cells. Through genome-wide RNAi screening in *Drosophila*, it was found that dNRAMP, the single *Drosophila* homolog of mammalian NRAMPs, is important for virus binding and entry (**Figure 9**). Overexpression of NRAMP2 increased the percentage of infected cells, while NRAMP2 deficiency rendered mammalian cells nonpermissive to SINV infection (Rose et al., 2011).

Another receptor, PHB1, was identified as a cellular receptor for CHIKV in human cells in 2012 (**Figure 8**) (Wintachai et al., 2012). It was observed to interact with the E2 protein of CHIKV under confocal microscopy. Using a two-dimensional virus overlay protein-binding assay, PHB1 was identified as one of the CHIKV-binding proteins. Experimental results showed a significant reduction in the number of infected cells in the presence of anti-PHB1 antibodies. Co-localization, co-immunoprecipitation, and antibody- and siRNA-mediated infection inhibition studies confirmed the interaction between PHB1 and the CHIKV E2 protein in human cells (**Figure 9**) (Wintachai et al., 2012). Interestingly, PHB1 also serves as a putative receptor for DENV-3 in SH-SY5Y and CHME-3 cells (Sharma et al., 2020).

**Figure 10:**
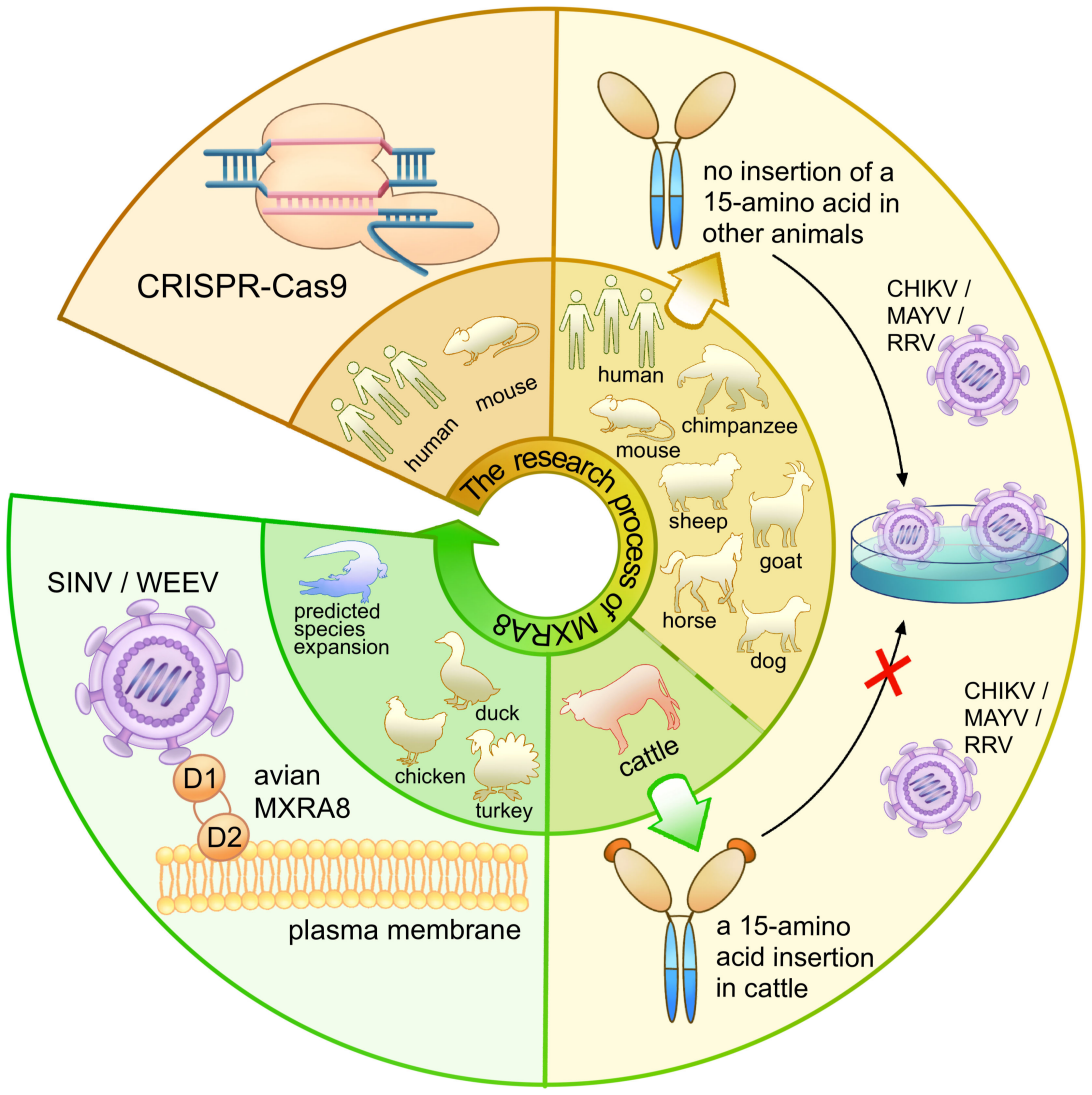
The research process and findings of MXRA8 as an alphavirus receptor. The diagram represents the research process for MXRA8, with the inner ring indicating the species of the receptor and the outer ring depicting the relevant experimental process. The brown panel shows that, through the use of CRISPR-Cas9, MXRA8 was identified as a receptor for multiple alphaviruses. The yellow panel presents the study of the extended host range of MXRA8 and the hosts (*Bovinae*) that cannot bind to the alphaviruses. The green panel illustrates the different binding modes of the avian MXRA8 binding to the alphaviruses.

In 2018, a significant breakthrough in the field of alphavirus receptor research coincided with the widespread use of gene editing in scientific research. CRISPR, a natural element of the bacterial immune system, has been widely harnessed by prokaryotes as a crucial survival mechanism for self-defense, enabling the degradation of invading plasmid DNA or bacteriophage DNA (Loureiro and da Silva, 2019). The introduction of the CRISPR/Cas9 system enabled the targeted modification, insertion, or replacement of specific nucleic acid sequences (Cong et al., 2013; Mali et al., 2013; Sternberg et al., 2014). Published in 2018 by Zhang et al., an article discovered that MXRA8 could play a crucial role in the entry of several alphaviruses in both humans and mice (**Figure 8**). Gene editing of mouse MXRA8 or human MXRA8 resulted in reduced viral infection levels in fibroblasts and skeletal muscle cells (**Figure 10**) (Zhang et al., 2018). Over the following years, as science and technology continued to advance, researchers gained a deeper understanding of the protein structure of MXRA8 from multiple perspectives. Several articles attempted to explain the molecular connection between the virus and its receptor.

MXRA8, a cell adhesion molecule composed of two Ig-like domains, binds to a surface-exposed region across the A and B domains of the CHIKV E2 protein (**Figure 10**) (Zhang et al., 2018; Basore et al., 2019; Song et al., 2019). Domain 1 is formed by the hinge region and domain 2 (Zhang et al., 2018). MXRA8 directly binds to CHIKV particles, enhancing virus attachment and internalization in humans. The determinants of the MXRA8-binding site in CHIKV are generally conserved among arthritogenic alphaviruses (44% conserved) but more divergent in encephalitic alphaviruses (14% conserved) (Basore et al., 2019).

Another publication in 2020 by Kim et al. (2020) expanded the range of species to include mice, rats, chimpanzees, dogs, horses, goats, sheep, and humans. Within the cattle MXRA8, a 15-amino acid insertion was identified in the C′–C″ loop of domain D1. This insertion prevented MXRA8 from binding to CHIKV, while other species did not contain this amino acid sequence insertion (**Figure 10**). Experimental results presented in the article showed divergent outcomes when the amino acids were inserted or removed from bovine animals or when the 15-amino acids were removed from other animals.

In addition, MXRA8 can act as a receptor for SINV, WEEV, and related alphaviruses in avian reservoirs (**Figure 8**). Structural analysis suggests that duck MXRA8 D1 contacts E1 of WEEV, while mammalian MXRA8 predominantly contacts CHIKV E2 (Zimmerman et al., 2023b). Interactions occur with the wrapped, intraspike, and interspike heterodimers. Duck MXRA8 D1 makes primary contact with WEEV E1 in four sites: the C–C′ loop, C″–D loop, D–E loop, and B–C connector (**Figure 10**) (Zimmerman et al., 2023b). When D1 of the duck is replaced with D1 of the mouse MXRA8, MXRA8 fails to bind to WEEV. Further research suggests that reptiles may also serve as potential future hosts (Zimmerman et al., 2023b).

Accompanied by the application of CRISPR-Cas9 in the field of alphavirus receptors, several receptors were subsequently discovered. In 2021, LDLRAD3, VLDLR, and ApoER2, members of the LDLR family, were identified as receptors for alphaviruses (**Figure 8**). The LDLRAD3 contains three LDLa repeats, which in other LDL receptor family members function to recognize ligands. LDLRAD3 also contains two conserved PPxY motifs, which can function as interaction motifs, and a conserved dileucine motif, which has been shown to mediate endocytosis in other proteins (Noyes et al., 2016). In 2020, Ma et al. (2020) found that gene editing of mouse Ldlrad3 or human LDLRAD3 resulted in highly reduced VEEV infection of neuronal cells. LDLRAD3 involves three LDL-receptor class-A domains involving D1, D2, and D3, with D1 predicted to interact with the VEEV as it is the farthest end of the plasma membrane. Injection of LDLRAD3(D1)-Fc can help mice against the infection of VEEV, further suggesting that D1 can interact with the virus (Ma et al., 2020). Both the antibodies for the LDLRAD3 and LDLRAD3-Fc fusion proteins block VEEV infection, which suggests a strategy for the prevention of severe VEEV infection (Ma et al., 2020). Two studies solved the structure of VEEV and LDLRAD3 in 2021, and domain 1 of LDLRAD3 is necessary and sufficient to support infection by VEEV (**Figure 8**) (Basore et al., 2021; Ma et al., 2021). The soluble decoy proteins with multiple LA3 repeats inhibit EEEV infection in mouse cell culture. LDLRAD3 was also identified as an ACE2-independent severe acute respiratory syndrome coronavirus 2 (SARS-CoV-2) receptor through genome-wide CRISPR activation screening (Zhu et al., 2022).

In 2021, Clark et al. (2022) found the E2 and E1 glycoproteins of SFV, EEEV, and SINV can interact with the ligand-binding domains of VLDLR and ApoER2. Under the use of CRISPR-Cas9, VLDLR and ApoER2 were identified as the entry receptors for EEEV/SFV/SINV. VLDLR contains an N-terminal ligand-binding domain with cysteine-rich repeats, a cluster of ECG modules containing a β-propeller domain, and a membrane-proximal *O*-linked sugar domain. The study found that the E2 and E1 glycoproteins of SFV, EEEV, and SINV interact with the ligand-binding domains of VLDLR and ApoER2 (Clark et al., 2022). The administration of a VLDLR LBD-Fc fusion protein can help against a rapidly fatal *Semliki Forest virus* infection in mice.

**Figure 11:**
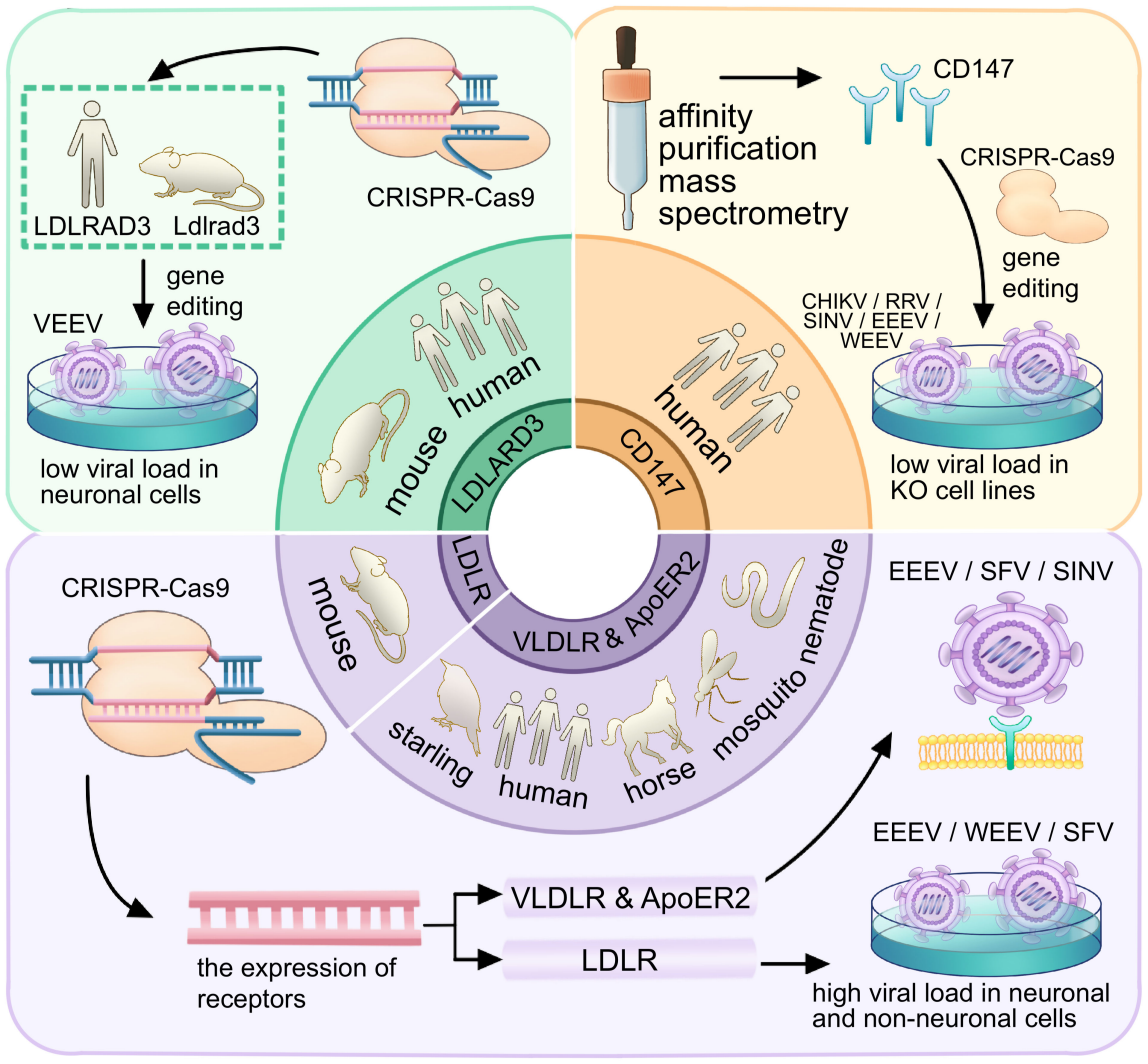
Receptor research process and experimental findings for CD147, LDLRAD3, LDLR, VLDLR, and ApoER2. The diagram represents the research process for CD147, LDLRAD3, LDLR, VLDLR, and ApoER2, with the inner ring indicating the species of the receptor and the outer ring depicting the relevant experimental process. The green panel shows that the use of CRISPR-Cas9 helped to identify LDLRAD3 (human) and LDLRAD 3 (mouse) as receptors for VEEV. The orange panel depicts that affinity purification mass spectrometry identified the CD147 as a receptor in humans. The purple panel demonstrates the identification of LDLR in mice and VLDLR & ApoER2 in starlings, humans, horses, mosquitoes, and nematodes using CRISPR-Cas9.

In 2024, Ma et al. (2024) performed a CRISPR-Cas9 genome-wide loss-of-function screen with the expression of the chimeric alphavirus expressing the EEEV structural proteins in mouse neuronal cells and identified LDLR as a low-affinity receptor for EEEV, WEEV, and SFV entry (**Figure 8**). Expression of LDLR on the surface of neuronal or non-neuronal cells can help facilitate the binding and infection of EEEV, WEEV, and SFV (**Figure 11**). LDLR is a membrane protein composed of seven LA repeats, two ECG-like domains, a beta-propeller domain, another ECG-like domain, and a stretch of residues with multiple *O*-linked glycosylation sites. LA domain proteins with three or five tandem repeats were designed and expressed in the context of an Fc-fusion protein, which helped to counteract the activity of EEEV and WEEV in cell culture (Ma et al., 2024).

CD147 acts as an alternative receptor for SARS-CoV-2 entry into cells with low or undetectable ACE2 expression (Lim et al., 2022). It is also an entry factor for various viruses, including human immunodeficiency virus (HIV) (Pushkarsky et al., 2001) and SARS-CoV (Chen et al., 2005). Additionally, it was discovered as a receptor for alphavirus in 2021 by De Caluwé et al. (2021) (**Figure 8**). It was found that affinity purification mass spectrometry can allow the identification of factors that facilitate entry of CHIKV in human cells, and it was further validated using CRISPR-Cas9 (De Caluwé et al., 2021). CD147 contains similar protein domains as another alphavirus receptor, MXRA8. The CD147 protein complex is involved in a step prior to viral RNA synthesis and can play an important role in the replication cycle of alphaviruses (**Figure 11**) (De Caluwé et al., 2021). In summary, many recent studies focused on the hosts of various alphavirus receptors, the structure of receptors, and their binding with alphaviruses. They employed a variety of methods to identify potential receptors and gain a more detailed understanding of them. They not only provide guidance and support for further exploration of alphavirus receptors in the future but also lay the groundwork for investigating receptors of other viruses.

## 4 Discussion

The study of virus receptors in the context of alphaviruses has significant implications for public health and clinical practice. Understanding the receptors through which viruses enter cells enables the development of targeted therapies and vaccines. By identifying specific receptors, such as MXRA8 or LDLRAD3, researchers can design interventions to block viral entry, potentially preventing infection or reducing its severity. Knowledge of virus receptors can also inform the development of diagnostic tools. For instance, detecting the expression levels of specific receptors in patient samples could aid in the early diagnosis or monitoring of viral infections. Identifying the receptors involved in viral transmission is crucial for assessing the risk of outbreaks and monitoring the spread of viruses within populations. This information guides public health interventions and surveillance efforts to prevent and control outbreaks effectively. Understanding the diversity of virus receptors across different species, as suggested in the case of MXRA8 and reptiles, may inform personalized approaches to treatment and prevention. This is particularly relevant in zoonotic infections, where viruses can jump between animal and human hosts. Insights into virus–receptor interactions can drive the development of novel antiviral drugs. By targeting specific receptors or blocking viral attachment, researchers can explore new avenues for therapeutic intervention against viral infections. Public health strategies aimed at preventing viral infections can benefit from knowledge of virus receptors. This includes measures such as promoting hygiene practices, vaccination campaigns, and environmental interventions to reduce the transmission of viruses through their respective receptors. Overall, the study of virus receptors has the potential to significantly impact public health efforts by informing the development of targeted interventions, enhancing diagnostic capabilities, and improving our understanding of viral pathogenesis and transmission dynamics.

Our study is the first to conduct a comprehensive bibliometric analysis of the alphavirus receptor from 2013 to 2024. We chose the Scopus database for this extraction as it contains a wide range of publications in the field of biomedicine. We have demonstrated that paper visualization methods can help us gain a deeper understanding of the data and identify potential foci within the field. By applying tools such as CiteSpace, VOS Viewer, Bibliometrix, and Scimago Graphica, we can create more visual figures and form a more intuitive impression of future development.

In this research, a total of 167 related articles were retrieved. Further analysis indicates that these articles were contributed by institutions, authors, and countries. The number of publications and betweenness centrality are vital indicators reflecting the overall development in the alphavirus receptor field in terms of country and author analysis. The author who published the most articles is Diamond (*n* = 26), and the most cited author is Weaver (*n* = 55). The USA leads in contributing to this field, as evidenced by its highest number of international collaborations and publications.

The trends in the study of alphavirus receptors correspond with the outbreak of alphaviruses during specific years. Additionally, flow cytometry, enzyme-linked immunosorbent assay, Vero cell line, tumor necrosis factor, glycosylation, and immune response are also trending in the field. The cluster containing the most nodes is related to sensing viral invasion. The word cloud highlights important terms such as alphavirus, nonhuman, and animal, emphasizing the significance of animal hosts and receptor species genes in the study of alphaviruses.

In subsequent analyses, we introduced several receptors related to alphaviruses individually, focusing on their research methods and processes. Gene editing technology, particularly CRISPR-Cas9, has been commonly used in discovering receptors such as MXRA8, LDLRAD3, LDLR, VLDLR, and ApoER2. Structural analysis and previous research have provided insights into the receptor structure, with specific parts playing unique roles. Animal models, particularly mammals like humans and mice, are commonly used in research. The timeline perspective of alphavirus receptors research shows a sequential progression based on previous studies, expanding to similar types of viruses. For example, LDLRAD3, VLDLR, and ApoER2, members of the LDLR family, were initially selected as receptors for alphaviruses, and subsequent research introduced a new receptor, LDLR. MXRA8 research started with humans and mice and gradually expanded to other mammals, with recent research considering avian species and reptiles as potential hosts.

Although this study is the first to utilize bibliometric methods and provide visual displays of publications on alphavirus receptors, it has several limitations. The Scopus database may not contain all previous publications, and while it is a commonly used literature research tool, it is not exhaustive. The tools we used also have limitations and may not provide accurate information or answers to existing research problems, which can impact the research process. Another limitation is that we did not thoroughly examine individual article records, except for those in a random sample we used to verify accuracy.

Future directions in alphavirus receptor research may emphasize expanding the host range of these receptors. Members of the LDLR family, such as LDLRAD3, VLDLR, ApoER2, and LDLR, play significant roles in the discovery of alphavirus receptors, suggesting that additional receptors within this family may yet be identified. The CRISPR-Cas9 technique has been instrumental in the research process for identifying alphavirus receptors, and it is anticipated that more advanced methodologies centered on alphavirus receptors will be employed.

## Data availability statement

The raw data supporting the conclusions of this article will be made available by the authors, without undue reservation.

## Author contributions

RC: Investigation, Software, Writing – original draft. ZW: Software, Writing – review & editing. LZ: Conceptualization, Writing – review & editing.
